# Supplementary material for: Whole genome comparisons reveal panmixia among fall armyworm (Spodoptera frugiperda) from diverse locations
Source: BMC Genomics. 2021 Mar 12;22:179. doi: 10.1186/s12864-021-07492-7 (PMC7953542; doi:10.1186/s12864-021-07492-7)
Supplement: Supplementary file 1 — Additional file 1: Supplementary Table 1. Describing details of samples analyzed, and Table 2 showing Mash distances based on country. Supplementary Figure S1. Showing clustering based on Mash distances, Figure S2 showing amplification of Wolbachia in selected samples, Figure S3 showing the amplified sequences in Fig. S2, and Figure S4 showing the geographic locations sampled. [file 12864_2021_7492_MOESM1_ESM.docx]

**Supplementary Tables and Figures**

Whole genome comparisons reveal panmixia among fall armyworm (*Spodoptera frugiperda*) from diverse locations

Katrina A. Schlum^1^, Kurt Lamour^2^, Caroline Placidi de Bortoli^2^, Rahul Banerjee^2^, Scott J. Emrich^1,3^, Robert Meagher^4^, Eliseu Pereira^5^, Maria Gabriela Murua^6^, Gregory A. Sword^7^, Ashley E. Tessnow^7^, Diego Viteri Dillon^8^, Angela M. Linares Ramirez^9^, Komivi S. Akutse^10^, Rebecca Schmidt-Jeffris^11^, Fangneng Huang^12^, Dominic Reisig^13^, and Juan Luis Jurat-Fuentes^2^

^1^Genome Science and Technology Graduate Program, University of Tennessee, Knoxville, TN 37996, USA

^2^Department of Entomology and Plant Pathology, University of Tennessee, Knoxville, TN 37996, USA

^3^Department of Electrical Engineering and Computer Science, University of Tennessee, Knoxville, TN, USA

^4^USDA-ARS Center for Medical, Agricultural and Veterinary Entomology (CMAVE), Insect Behavior and Biocontrol Research Unit, Gainesville, FL 32608, USA

^5^Departamento de Entomologia, Universidade Federal de Viçosa, Viçosa, MG 36570, Brazil

^6^Estación Experimental Agroindustrial Obispo Colombres, Las Talitas, Tucumán T4101XAC, Argentina

^7^Department of Entomology, Texas A&M University, College Station, TX 77843, USA

^8^Isabel Research Substation, Department of Agro-Environmental Sciences, University of Puerto Rico, Isabela, PR 00662, USA

^9^Lajas Research Substation, Department of Agro-Environmental Sciences, University of Puerto Rico, Lajas, PR 00667, USA

^10^International Centre of Insect Physiology and Ecology, Nairobi, Kenya

^11^USDA-ARS Temperate Fruit & Vegetable Research Unit, Wapato, WA 98951, USA

^12^Department of Entomology, Louisiana State University Agricultural Center, Baton Rouge, LA 70803, USA

^13^Department of Entomology and Plant Pathology, North Carolina State University, Raleigh, NC 27695, USA

**Materials and Methods**

**Detection of *Wolbachia***

Total genomic DNA was extracted from the abdomen of individual *Spodoptera frugiperda* moths using the Blood and Tissue DNEasy Kit (Qiagen) following the manufacturer’s protocol. Primers to amplify the *Wolbachia* surface protein (*wsp*) marker (Zhou et al., 1998) were forward *wsp* 81F (5’-TGGTCCAATAAGTGATGAAGAAAC-3’) and reverse *wsp* 691R (5’-AAAAATTAAACGCTACTCCA-3’). Reactions included 2x PCR Master Mix (Invitrogen^TM^ Platinum^TM^ SuperFi^TM^), 1 µM of each primer, 10 ng of gDNA and nuclease-free water to 25 µl of final volume. Amplification conditions in an Eppendorf Mastercycler Ep Gradient Thermal Cycler included initial denaturation at 98°C for 30 sec, followed by 35 cycles of denaturation at 98° for 10 sec, annealing at 55.9°C for 10 sec and extension at 72°C for 30 sec, finalized with a final extension step at 72°C for 30 sec. A positive control consisting of *Wolbachia pipientis* gDNA provided by The Wolbachia Project (Vanderbilt University, TN, USA) was included in all experiments. Amplicons were checked through 1% agarose gel electrophoresis, purified and sequenced at the Sequencing Core facility at the University of Tennessee (Knoxville).

**Supplementary Tables**

**Supplementary Table 1** - List of *Spodoptera frugiperda* samples sequenced and used in this work. Shown is information on the location where collected (or facility for laboratory reference strains), Cry1F resistance phenotype (u for unknown, s for susceptible, r for resistant), collection (F for field-collected, L for lab-reared), host strain based on *Tpi*183 marker (corn, rice, hybrid or unknown), and the stage used for genomic DNA purification.

| **Sample Name** | **Country** | **City/County/Facility** | **Municipality/State originally collected** | **Cry1F** | **Collection** | **Host strain** | **Stage** |
| --- | --- | --- | --- | --- | --- | --- | --- |
| ArgBAu1 | Argentina | Buenos Aires | Buenos Aires | u | F | Corn | Moth |
| ArgJs1 | Argentina | Estacion Experimental Agroindustrial Obispo Colombres (EEAOC, Tucuman) | Humahuaca (Jujuy) | s | L | Corn | Moth |
| ArgSFu1 | Argentina | San Justo | Santa Fe | u | F | Corn | Moth |
| ArgXXr1 | Argentina | Overo Pozo | Tucuman | r | L | Corn | Moth |
| ArgXXr2 | Argentina | La Cocha | Tucuman | r | L | Corn | Moth |
| BraBAr1 | Brazil | Luís Eduardo Magalhães | Bahia | r | L | Corn | Larva |
| BraBAr2 | Brazil | Luís Eduardo Magalhães | Bahia | r | L | Corn | Larva |
| BraBAr3 | Brazil | Luís Eduardo Magalhães | Bahia | r | L | Corn | Larva |
| BraBAr4 | Brazil | Luís Eduardo Magalhães | Bahia | r | L | Corn | Larva |
| BraBAr5 | Brazil | Luís Eduardo Magalhães | Bahia | r | L | Corn | Larva |
| BraMGr1 | Brazil | Viçosa | Minas Gerais | r | L | Corn | Larva |
| BraMGr2 | Brazil | Viçosa | Minas Gerais | r | L | Corn | Larva |
| BraMGr3 | Brazil | Viçosa | Minas Gerais | r | L | Corn | Larva |
| BraMGr4 | Brazil | Viçosa | Minas Gerais | r | L | Corn | Larva |
| BraMGr5 | Brazil | Viçosa | Minas Gerais | r | L | Corn | Larva |
| BraSPr1 | Brazil | Casa Branca | Sao Paulo | r | L | Corn | Larva |
| BraSPr2 | Brazil | Casa Branca | Sao Paulo | r | L | Unknown | Larva |
| BraSPr3 | Brazil | Casa Branca | Sao Paulo | r | L | Corn | Larva |
| KenXXu1 | Kenya | International Centre of Insect Physiology and Ecology (ICIPE, Nairobi) | Siaya and Homa Bay counties | u | L | Corn | Moth |
| KenXXu2 | Kenya | International Centre of Insect Physiology and Ecology (ICIPE, Nairobi) | Siaya and Homa Bay counties | u | L | Corn | Moth |
| KenXXu3 | Kenya | International Centre of Insect Physiology and Ecology (ICIPE, Nairobi) | Siaya and Homa Bay counties | u | L | Corn | Moth |
| PueGUr1 | Puerto Rico |  | Guayama | r | F | Corn | Larva |
| PueLAu1 | Puerto Rico |  | Lajas | u | F | Corn | Moth |
| PueSIu1 | Puerto Rico |  | Santa Isabel | u | F | Rice | Moth |
| USAFLr1 | USA | Collier County | Florida | r | L | Corn | Larva |
| USAFLr2 | USA | Collier County | Florida | r | L | Corn | Larva |
| USAFLu1 | USA | Belle Glade (Palm Beach County) | Florida | u | F | Corn | Moth |
| USAFLu10 | USA | Hague (Alachua County) | Florida | u | F | Corn | Moth |
| USAFLu11 | USA | Hague (Alachua County) | Florida | u | F | Corn | Moth |
| USAFLu12 | USA | Hague (Alachua County) | Florida | u | F | Corn | Moth |
| USAFLu2 | USA | Belle Glade (Palm Beach County) | Florida | u | F | Rice | Moth |
| USAFLu3 | USA | Miami (Miami-Dade County) | Florida | u | F | Corn | Moth |
| USAFLu4 | USA | Miami (Miami-Dade County) | Florida | u | F | Corn | Moth |
| USAFLu5 | USA | Miami (Miami-Dade County) | Florida | u | F | Hybrid | Moth |
| USAFLu6 | USA | Miami (Miami-Dade County) | Florida | u | F | Corn | Moth |
| USAFLu7 | USA | Miami (Miami-Dade County) | Florida | u | F | Corn | Moth |
| USAFLu8 | USA | Hague (Alachua County) | Florida | u | F | Corn | Moth |
| USAFLu9 | USA | Hague (Alachua County) | Florida | u | F | Corn | Moth |
| USAMDu1 | USA | Jarretsville (Harford County) | Maryland | u | F | Rice | Moth |
| USAMDu2 | USA | Jarretsville (Harford County) | Maryland | u | F | Rice | Moth |
| USAMNu1 | USA | Rosemount (Dakota County) | Minnesota | u | F | Corn | Moth |
| USAMNu2 | USA | Rosemount (Dakota County) | Minnesota | u | F | Corn | Moth |
| USAMSs1 | USA | Benzon Research Inc (Carlisle, PA) | Mississippi | s | L | Corn | Moth |
| USAMSs2 | USA | USDA-ARS Southern Insect Management Reseacrh Unit (SIMRU, Stoneville) | Mississippi | s | L | Corn | Moth |
| USANCr1 | USA | Hyde County | North Carolina | r | L | Corn | Moth |
| USASCu1 | USA | Charleston (Charleston County) | South Carolina | u | F | Corn | Moth |
| USASCu2 | USA | Charleston (Charleston County) | South Carolina | u | F | Corn | Moth |
| USATNu1 | USA | Crossville (Cumberland County) | Tennessee | u | F | Rice | Moth |
| USATNu2 | USA | Crossville (Cumberland County) | Tennessee | u | F | Rice | Moth |
| USATXu1 | USA | Corpus Christi (Nueces County) | Texas | u | F | Unknown | Moth |
| USATXu2 | USA | College Station (Brazos County) | Texas | u | F | Rice | Moth |
| USATXu3 | USA | College Station (Brazos County) | Texas | u | F | Rice | Moth |
| USATXu4 | USA | Corpus Christi (Nueces County) | Texas | u | F | Hybrid | Moth |
| USATXu5 | USA | Lubbock (Lubbock County) | Texas | u | F | Hybrid | Moth |
| USATXu6 | USA | Lubbock (Lubbock County) | Texas | u | F | Corn | Moth |

**Supplementary Table 2 -** Average Mash distances based on country of origin based on all 55 samples

| **Country of origin** | **Argentina** | **Brazil** | **Kenya** | **Puerto Rico** | **USA** |
| --- | --- | --- | --- | --- | --- |
| **Argentina** |  | 0.043 | 0.044 | 0.044 | 0.044 |
| **Brazil** | 0.043 |  | 0.043 | 0.045 | 0.045 |
| **Kenya** | 0.044 | 0.043 |  | 0.043 | 0.043 |
| **Puerto Rico** | 0.044 | 0.045 | 0.043 |  | 0.045 |
| **USA** | 0.044 | 0.045 | 0.043 | 0.045 |  |

**Supplementary figures**

**Figure S1**: Complete clustering on Mash distances heatmap of Mash distances across 55 samples colored by host strain and rooted with *Spodoptera litura*.


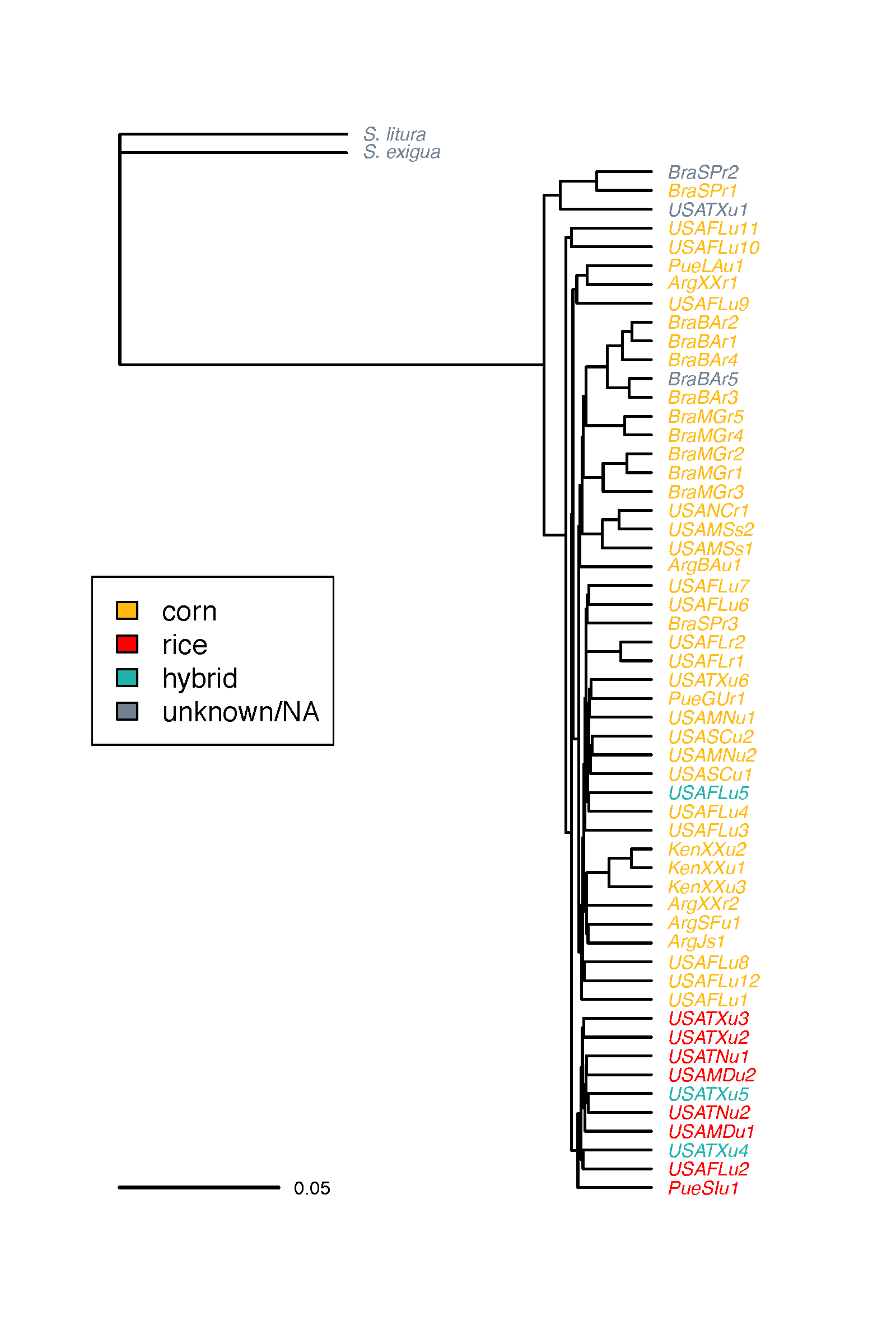


**Figure S2**: Amplification of a fragment of the *wsp* gene (610 bp) from *S. frugiperda* moths. A positive control (*Wolbachia pipiens*) was also included (+C). Samples shown ArgXXr2 (lane 1), PueSIu1 (lane 2), USAFLu8 (lane 3), USAFLu10 (lane 4), USAFLu12 (lane 5), USAMDu2 (lane 6), USATXu5 (lane 7) and *W. pipiens* (lane 8). Sequenced amplicons matched with 99.8% identity to *Wolbachia* endosymbiont of *Nasonia vitripennis* seqvar1 Nvit outer surface protein (*wsp*) gene (GenBank accession number DQ380865).


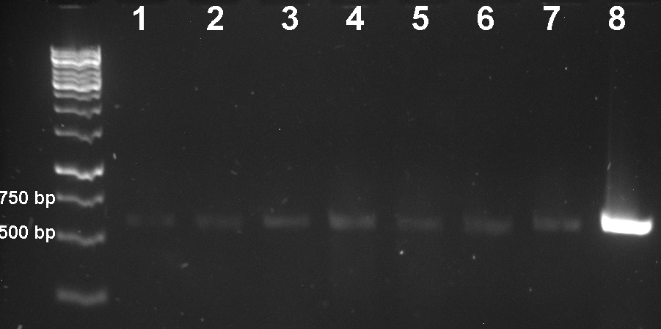


**Figure S3**: Sequence (listed 5’ to 3’) of amplicon obtained as described in Fig. S2 from samples USAMDu2 and USAFLu8 (belonging to different mitochondrial lineage clusters in Fig. 5). In BLASTn searches of the NCBInr *Wolbachia* database, this amplicon sequence was identical to the *wsp* gene of the *Wolbachia* endosymbiont from *Linognathus africanus* strain wLaf (accession number AY330317.1).

TGGTCCAATAAGTGATGAAGAAACTAGCTACTACGTTCGTTTGCAATACAACGGTGAAATTTTACCTCTTTTCACAAAAGTTGATGGTATTACCTATAAGAAAGGCAATAGTGATTACAGTCCATTAAAAGCGTCTTTTATAGCTGGTGGTGGTGCGTTTGGTTACAAAATGGACGACATCAGGGTTGATGTTGAAGGAGTTTATTCATACCTAAACAAAAATGATGTTACAGATGCAAAATTTACGCCAGATGCTATTGCAGACAGTTTAACAGCAATTTCAGGACTAGTTAACGTTTATTACGATATAGCAATTGAAGATATGCCTATCACTCCATATATTGGTGTTGGCGTTGGTGCAGCGTATATTAGCACACCTTTGGCAACTGCTGTGAGTAGTCAAAATGGTAAATTTGCTTTTGCTGGTCAAGCAAGAGCTGGTGTTAGTTACGATGTAACTCCAGAAGTCAAACTTTACGCTGGAGCTCGCTATTTCGGTTCTTATGGTGCTAACTTTGATAAAACTGACAAAGACGGCAAAGGGGAACTCAAAGTTCTTTACAGCACTGTTGGTGCAGAAGCTGGAGTAGCGTTTAATTTTT

**Figure S4**: Geographical distribution based on all the 55 *S. frugiperda* samples used in the study with dots proportional to the number of samples collected for each location.


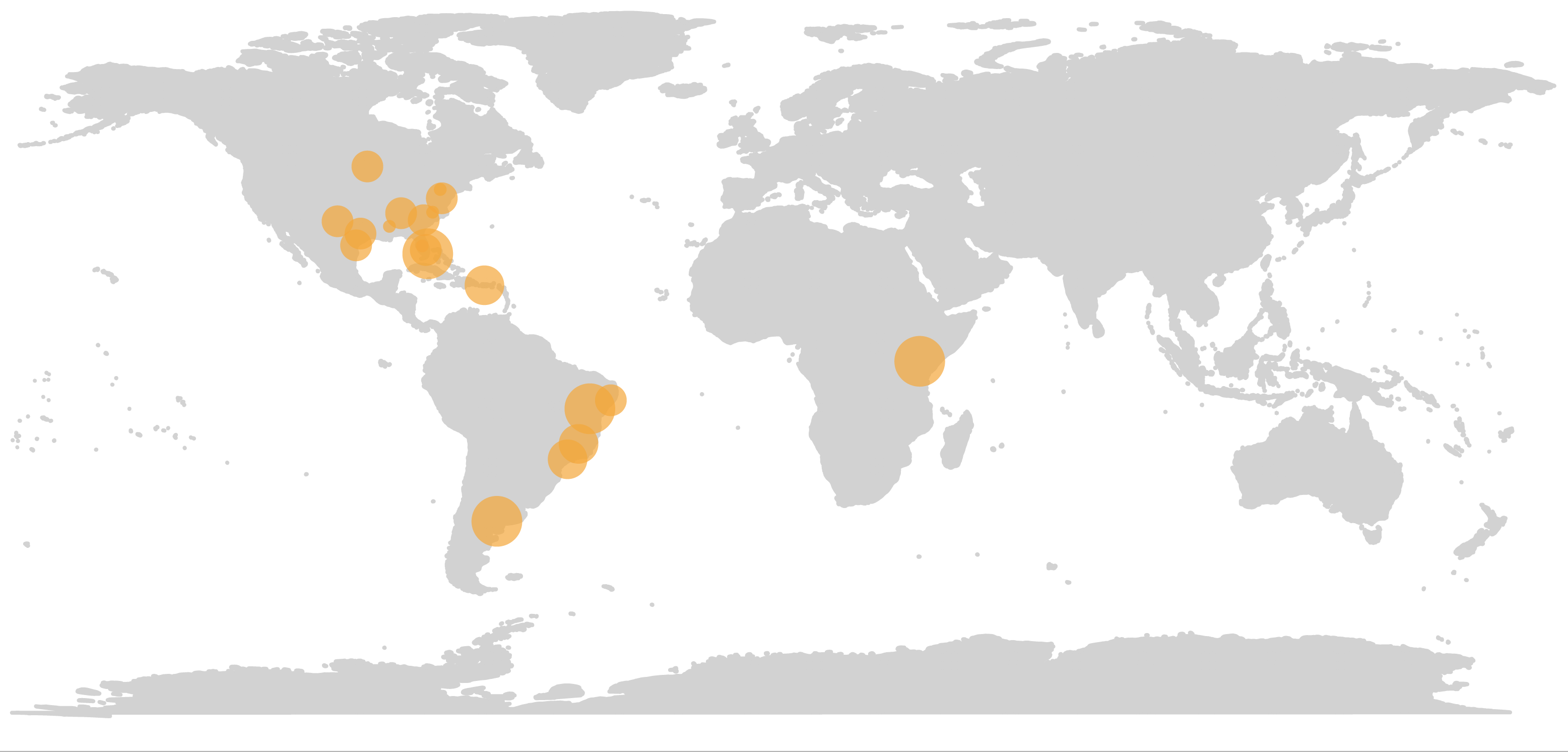


**References**

Zhou, W.G., Rousset, F. and O'Neill, S. 1998. Phylogeny and PCR-based classification of *Wolbachia* strains using *wsp* gene sequences. *Proceedings of the Royal Society B-Biological Sciences* 265**,** 509-515.
